# The Systemic Immune-Inflammation Index and the Risk of Parkinson’s Disease in the U.S.: A Cross-Sectional Study

**DOI:** 10.3390/jcm14020403

**Published:** 2025-01-10

**Authors:** Fujun Liu, Qibo Ran, Huajin Zhang, Jing Chen

**Affiliations:** 1 State Key Laboratory of Biotherapy Center, West China Hospital, Sichuan University, Chengdu 610041, China; lfj2021@scu.edu.cn (F.L.); zhanghuajin@stu.scu.edu.cn (H.Z.); 2 Department of Ophthalmology, West China Hospital, Sichuan University, Chengdu 610041, China; 3 Department of Neurosurgery and Neuromodulation Center, West China Hospital, Sichuan University, Chengdu 610041, China

**Keywords:** Parkinson’s disease, systemic immune-inflammation index, neurodegeneration, cross-sectional study, NHANES

## Abstract

**Background**: Inflammation is reportedly related to Parkinson’s disease (PD). However, the relationship between the systemic immune-inflammation index (SII) and PD remains unexplored. This study aimed to explore the potential relationship between the SII and PD. **Methods**: This retrospective cross-sectional study analyzed data from the National Health and Nutrition Examination Survey (NHANES) covering the years 2003 to 2020. We analyzed patients over 40 years of age after excluding those with missing SII, PD and covariate data. Logistic regression, subgroup analysis, and restricted cubic spline models were subsequently conducted to evaluate the associations between the SII and PD. **Results**: Finally, 30,638 participants were included in this study, of whom 416 (1.36%) were identified as having PD. Weighted multivariate regression analysis, adjusted for all covariates, revealed that participants with elevated in-transform (SII) values had a higher likelihood of PD [OR 1.39; 95% CI (1.02, 1.91), *p* = 0.039] compared to those with lower SII values. The fully adjusted restricted cubic spline curve revealed that the SII/100 was positively and linearly associated with the incidence of PD (*p* for nonlinearity > 0.05). Additionally, subgroup analysis revealed a stronger correlation between the SII and PD in female participants [OR = 1.06, 95% CI (1.03, 1.08)] compared to male participants [OR = 1.02, 95% CI (1.00, 1.03)] (*p* for interaction = 0.01). **Conclusions**: The SII showed a positive correlation with the incidence of PD, particularly in females. Further large-scale prospective studies are necessary to confirm these findings and explore the causal factors that may contribute to the early prevention of PD.

## 1. Introduction

Parkinson’s disease (PD) is a complex, progressive neurodegenerative disorder characterized by diverse motor and nonmotor symptoms that significantly impair functionality, restrict daily independence, and adversely affect mental health and quality of life in elderly individuals [1,2]. The primary pathological features of PD include the degeneration of dopamine-producing neurons and the formation of Lewy bodies in the substantia nigra [3]. Notably, the majority of substantia nigra dopamine neurons are lost before the primary symptoms begin to manifest [4]. Therefore, the early detection of PD via sensitive and specific biomarkers before symptom onset is urgently needed. Furthermore, PD is the second most prevalent neurodegenerative disorder, affecting approximately 0.3% of individuals over 40 years of age, and it poses a significant global public health challenge [5,6]. The global aging population is expected to lead to a more than 30% increase in the prevalence and incidence of PD by 2030 [7]. This will impose immense economic burdens on families, society, and medical service systems. Thus, PD should be taken seriously.

Neuroinflammation is a prevalent and key component of PD [8,9,10]. Chronic microglial activation, immune cells in the brain with macrophage-like properties, contributes significantly to neuronal loss [11]. Microglia release pro-inflammatory cytokines like IL-1β and TNF-α, along with reactive oxygen species, which exacerbate dopaminergic neuron degeneration, compromise the blood–brain barrier (BBB), and permit peripheral immune cells to infiltrate, thereby intensifying neuroinflammation [11]. Furthermore, for progressive supranuclear palsy—parkinsonism predominant, microglial activation may also play a similar role, potentially leading to midbrain atrophy and structural changes visible on neuroimaging [12]. Peripheral inflammatory changes during PD involve neutrophil hyperactivation and elevated lymphocyte infiltration into the central nervous system (CNS) through the compromised BBB [13]. These peripheral inflammatory markers contribute to CNS dysfunction. Given the critical role of inflammation in PD, recent research has increasingly focused on hematological biomarkers to assess the risk of inflammation.

The neutrophil-to-lymphocyte ratio (NLR), granulocyte-to-lymphocyte ratio (GLR), platelet-to-lymphocyte ratio (PLR), and systemic immune-inflammation index (SII) are widely recognized, cost-effective, and common biomarkers for assessing peripheral systemic inflammation [14,15]. Several studies indicate that the elevated NLR can reflect the degree of dopaminergic degeneration and reduced striatal dopamine transporter levels in patients with PD [16,17]. PLR may reflect the degree of BBB destruction and correlate with the severity of the disease [18]. Furthermore, the GLR, PLR, and NLR, along with C-reactive protein, can predict PD onset [8,19]; however, studies on the SII in patients with PD are lacking, and the associations between the SII and PD remain unclear.

The SII is a novel biomarker reflecting patients’ inflammatory and immune status by integrating neutrophils, lymphocytes, and platelets from peripheral blood [20]. The SII was initially employed to forecast the outcomes of different tumors [21]. It is now regarded as a sensitive and reliable marker for accurately assessing inflammatory and immune status [22]. Because the SII integrates neutrophil, platelet, and lymphocyte counts, it provides a broader perspective on immune–inflammatory balance. In addition, SII can serve as a more precise marker of overall inflammatory status when compared to both the PLR and the NLR [15].

This study aims to explore the relationship between the SII and PD risk based on prior evidence linking systemic inflammation with neurodegenerative processes [23]. As a readily accessible marker from routine blood tests, it offers a scalable approach to exploring its potential role in predicting PD risk and progression. Investigating the SII in PD may uncover novel links between systemic inflammation and neurodegenerative processes. This study utilized data from the National Health and Nutrition Examination Survey (NHANES) spanning 2003 to 2020 to evaluate the association between the SII and PD risk among U.S. adults. While we hypothesize that an elevated SII is associated with increased PD risk, this study does not aim to establish causality. Instead, the findings provide preliminary evidence to guide future longitudinal studies that could examine whether systemic inflammation directly contributes to PD pathogenesis or serves as a secondary response to neurodegenerative processes.

## 2. Patients and Methods

### 2.1. Study Design and Population

The NHANES is a cross-sectional U.S. survey employing a stratified multistage probability sampling method to monitor the nation’s health and nutritional status, providing critical data to policymakers, researchers, and the public. The survey data were gathered through home visits, screenings, and laboratory tests conducted at a mobile examination center (MEC). Survey data are released biennially. The NHANES data are publicly accessible at https://www.cdc.gov/nchs/nhanes/ (accessed on 1 December 2024). All participants provided written informed consent as approved by the NCHS Ethics Review Committee. The secondary analysis was exempt from additional Institutional Review Board approval. We analyzed data from nine to two years of NHANES cycles spanning from 2003 to 2020. Study participants, over 40 years old underwent interviews and assessments at the MEC. In addition, participants with missing data on PD, SII values, or covariates were excluded from this study. The final sample size used in our analysis was 30,638.

### 2.2. Exposure and Outcome Definitions

In this study, the SII was designed as the exposure variable, and it is defined as the neutrophil count/lymphocyte count × platelet count. Peripheral blood samples were analyzed through Beckman Coulter HMX hematology analyzers to measure the lymphocytes, neutrophils, and platelet counts, reported as 10^3^ cells/mL. The outcome variable was set as whether a PD diagnosis was present. The PD diagnosis was determined based on participants’ use of one or more anti-PD medications, as indicated by their responses to a prescription medication questionnaire. The individuals were classified as having PD if they responded affirmatively. Individuals who did not indicate that they were taking any anti-PD medications were classified as not having PD.

### 2.3. Covariates

Based on the literature and medical expertise, we included potential covariates that could confound the relationship between the SII and PD in this study. We considered demographic factors including sex, age, race (non-Hispanic white, non-Hispanic black, other races), education level (less than high school, high school, more than high school), and marital status (married or unmarried), along with smoking status (nonactive or active smoker) and various biological markers, such as white and red blood cell counts; alanine and aspartate aminotransferase levels; and globulin, creatinine, uric acid, sodium, and calcium levels. Additionally, chronic medical conditions, such as hypertension, hyperlipidemia, diabetes, coronary heart disease (CHD), and stroke, were included. The identification of chronic medical diseases was based on participants’ self-reported information regarding prior medical diagnoses.

### 2.4. Statistical Analysis

Statistical analyses were performed using R version 4.2.2. Continuous variables are expressed as weighted means ± standard deviations, while categorical variables are shown as frequencies and percentages. Baseline characteristics were compared using t-tests and chi-square tests. Logistic regression analyses, both univariate and multivariate, were conducted to evaluate the associations between the SII and PD incidence. In multivariate logistic regression, the SII was analyzed as both a continuous variable and as a tertile. The crude model had no covariates. Model 1 was adjusted for age, sex, race, education level, and marital status, whereas Model 2 included these variables along with additional covariates: hypertension, hyperlipidemia, CHD, stroke, diabetes, and smoking status. Model 3 was adjusted for all covariates. Model 3 employed restricted cubic spline analysis to assess the potential nonlinear relationship between SII and PD incidence.

Additionally, subgroup analyses assessed the association between the SII and PD incidence across different stratifying factors, including age (<65 years, ≥65 years), sex (male, female), education level (less than high school, high school, more than high school), medical history (stroke, hyperlipidemia, diabetes, hypertension, CHD), and marital status (married, unmarried). Interaction *p*-values were calculated to assess the moderating effects of these stratifying factors on the relationship between the SII and PD incidence. The results are presented as odds ratios (ORs) with 95% confidence intervals (CIs). A two-sided *p*-value of 0.05 or below was deemed statistically significant. We applied a weighting method to reduce the dataset’s volatility.

## 3. Results

### 3.1. The Characteristics of the Study Population

**Figure 1:**
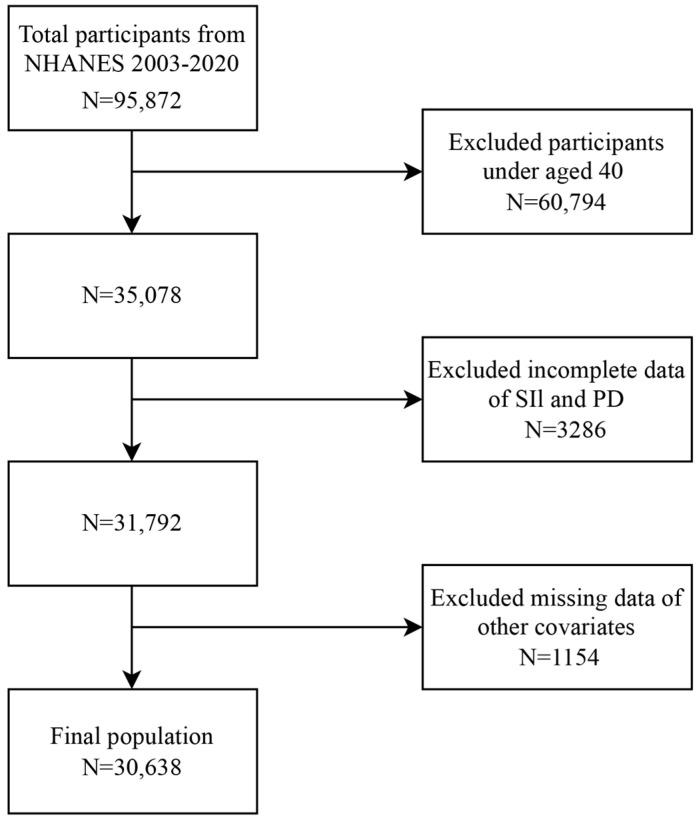
Flow diagram illustrating participant selection. PD, Parkinson’s disease; SII, systemic immune—inflammation index.

A total of 95,872 participants completed the interview, with 60,794 being under 40 years old. Subjects whose SII and PD data were missing (n = 3286) were excluded. We also excluded participants lacking data on other covariates (n = 1154). Finally, in total, 30,638 participants from the NHANES (2003–2020) were included in this cross-sectional study. The detailed process of inclusion and exclusion is illustrated in Figure 1.

Table 1 presents a comprehensive summary of the clinical and biochemical characteristics of the participants, stratified by PD. Among the 30,638 participants analyzed in the study, 15,624 (52.72%) were female and 15,014 (47.28%) were male, with a mean age of 58.34 ± 0.14 years. In our research, 416participants (1.36%) were classified as having PD. PD presence was significantly associated with age, sex, marital status, race, stroke status, diabetes status, CHD status, hypertension status, the SII, and blood cell count (all *p* < 0.05). In contrast, the trends for education level, smoking status, hyperlipidemia, serum globulin, uric acid, creatinine, alanine aminotransferase, aspartate aminotransferase, sodium, and calcium levels were not significant (*p* > 0.05). Compared with participants without PD, participants with PD tended to be older, unmarried, and White, and with a history of stroke, diabetes, CHD, and hypertension. Individuals with PD exhibited elevated SII values and white blood cell counts, alongside reduced red blood cell counts, compared with non-PD individuals.

### 3.2. Relationship Between the SII and PD

Table 2 presents the relationships between PD and various factors, including age, sex, race, marital status, education level, history of stroke, hyperlipidemia, diabetes, CHD, hypertension, smoking status, the SII, red blood cell counts, white blood cell counts, and multiple biochemical markers. As no significant effect values were observed, the SII was divided by 100 to amplify the effect values.

Using multifactorial logistic regression analysis, four models were constructed to explore the relationship between the SII/100 and PD incidence (Table 3). A significant positive association was observed between SII/100 and the incidence of PD. The SII/100 was analyzed as a continuous variable in the models. The unadjusted crude model revealed an OR of 1.07 (95% CI: 1.04, 1.10; *p* < 0.0001), suggesting a 7% higher probability of PD per unit increase in SII/100. Model 1 was adjusted by age, gender, ethnicity, educational level, and marital status. Model 2 was further adjusted by hypertension, hyperlipidemia, CHD, stroke, diabetes and smoking status. Model 3 was adjusted by all covariates included in this study. Model 1 [OR 1.06; 95% CI (1.03, 1.09), *p* < 0.001], Model 2 [OR 1.06; 95% CI (1.02, 1.09), *p* < 0.001], and Model 3 [OR 1.05; 95% CI (1.02, 1.09), *p* < 0.001] exhibited similar trends.

Sensitivity analysis was conducted for the SII/100 quartiles. The fully adjusted model for the SII/100 tertile revealed a consistent positive association between elevated SII values and higher odds of developing PD. Specifically, participants in Quartile 3 exhibited a 39% higher risk of developing PD compared to those in Quartile 1 [OR 1.39; 95% CI (1.02, 1.91), *p* = 0.039]. Furthermore, participants in Quartile 2 also had a greater risk of PD than did those in Quartile 1, but this relationship was not statistically significant [OR 1.29; 95% CI (0.87, 1.91), *p* = 0.2]. Moreover, all four models exhibited statistically significant trend *p*-values (*p* < 0.05).

**Figure 2:**
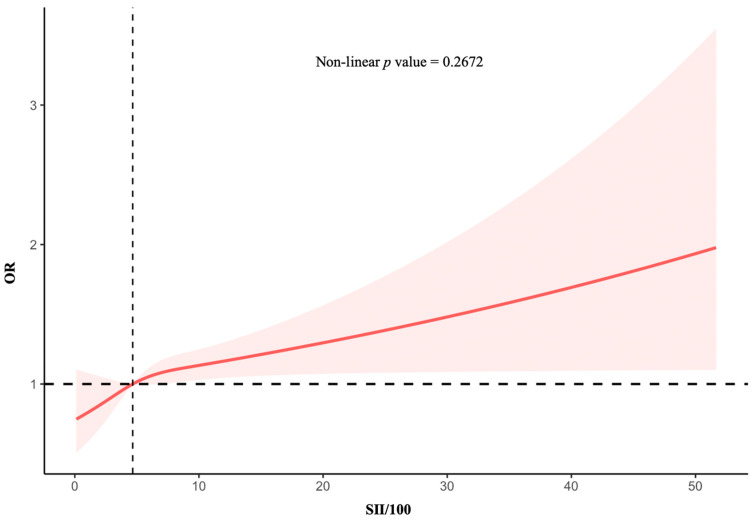
The restricted cubic spline plot describes the association between the SII/100 and the incidence of PD. The solid red line represents the smooth curve of fit between variables. The pink area represents the 95% confidence interval from the fit. This was adjusted for all covariates from Table 3. SII, systemic immune-inflammation index; OR odds ratio.

A restricted cubic spline was applied to evaluate the potential nonlinear correlations between the SII and PD risk. The fully adjusted analyses indicated a linear correlation between the SII/100 and PD incidence (*p* for nonlinearity > 0.05). The OR curve for PD initially increased steeply with increasing SII, followed by a plateau (Figure 2).

### 3.3. Subgroup Analyses

**Figure 3:**
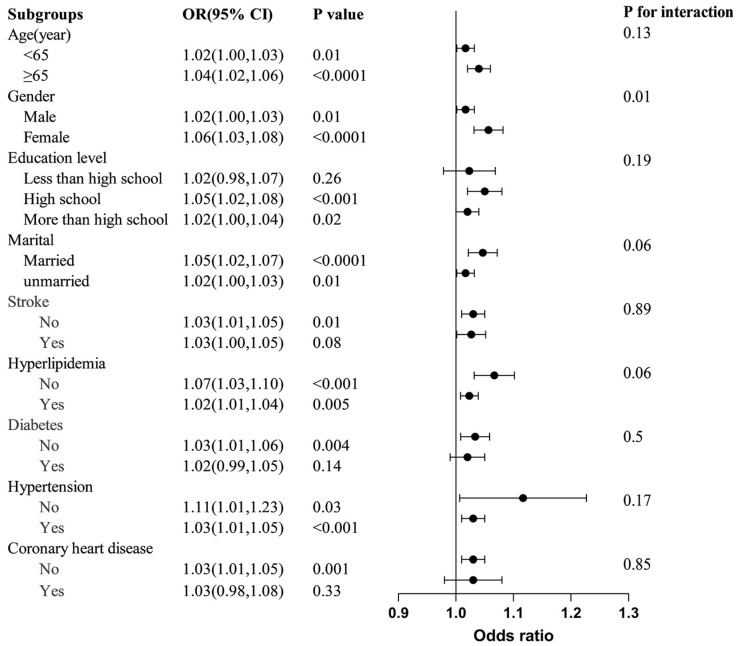
Subgroup analysis of the relationship between the SII and PD. OR, odds ratio; CI, confidence interval.

Subgroup analyses and interaction tests were performed to evaluate the associations between the SII and PD among different population subgroups stratified by age, sex, educational level, marital status, stroke, hyperlipidemia, diabetes, hypertension, and CHD (Figure 3). These subgroups subsequently underwent another round of analysis via a logistic regression model. In the subgroups categorized by age, sex, marital status, and hypertension status, the SII showed a significant association with PD across (all *p* < 0.05). For the subgroup stratified by stroke, diabetes, and CHD, significant correlations were observed only among the participants without these conditions. Moreover, a positive yet statistically insignificant correlation was identified between the SII and PD in participants with stroke, diabetes, and CHD, with OR values of 1.03, 1.02, and 1.03, respectively. The interaction test indicated no significant differences in the relationships between the SII and PD incidence across various factors, including age, educational level, marital status, stroke, hyperlipidemia, diabetes, and hypertension. These factors did not significantly influence the positive relationship, as all interaction *p*-values exceeded 0.05. However, there were notable interactions in the sex subgroups. Specifically, the correlation between the SII and PD was stronger in females (OR = 1.06) than in males (OR = 1.02).

## 4. Discussion

Recent studies have increasingly focused on the inflammatory response’s role in the progression and development of PD. In our study, we examined the relationship between the SII and PD incidence via a nationally representative sample of U.S. adults. Multivariate logistic regression analysis revealed that the SII was positively linked to the likelihood of developing PD after adjusting for all covariates. Additionally, the restricted cubic spline curve revealed that the SII/100 was positively and linearly associated with the incidence of PD. Subgroup analysis and interaction tests confirmed a significant relationship between the SII and PD in female participants. To the best of our understanding, this is the first study to investigate the relationship between the SII and the risk of developing PD.

PD incidence and related disability and mortality are rapidly increasing worldwide [24]. Many studies have shown that inflammatory indicators are correlated with the development and prognosis of PD in patients. However, the connection between the SII and PD incidence remains unclear. Lisanne et al. reported that people with an elevated adapted SII were found to have a higher likelihood of prevalent PD [8]. However, in their study, owing to the absence of neutrophil counts, they calculated the adapted SII as an exposure, which was calculated as the GLR times the platelet count. Therefore, the adapted SII is different from the SII in our study. In a study by Li et al. involving 148 consecutive patients with idiopathic PD, a higher SII score was associated with more severe motor impairments, suggesting that the SII may predict motor performance status in PD patients [25]. Our investigation revealed a potential association between elevated SII values and an increased likelihood of developing PD.

Current research on the inflammatory factors affecting PD has focused mainly on the NLR, C-reactive protein, immune cells, and cytokines. Recently, Li et al. reported that an elevated NLR and lymphocyte-to-monocyte ratio could be used for evaluating the severity of PD and that these inflammatory factors have potential as promising biomarkers for disease status [14]. One study by Liu et al. also revealed that the NLR was a risk factor for the progression of PD [26]. Additionally, C-reactive protein serves as a blood test indicator of bodily inflammation. Elevated hypersensitive C-reactive protein levels are significantly greater in PD patients than in normal controls, indicating its potential as a predictive biomarker for PD incidence [19]. Another study confirmed that hypersensitive C-reactive protein levels may serve as biomarker for the diagnose of PD [27]. Interestingly, some studies have demonstrated that the levels of CD8+ T cells, Tregs, and TNF-α are linked to cognitive impairment in PD patients and could act as potential markers for PD onset [16,28]. Additionally, IL-6 and TNF-α levels may also predict PD development [28]. Our findings also support a positive correlation between inflammation levels and PD risk, which aligns with the majority of existing research.

The pathological changes observed in PD are similar to those observed in autoimmune diseases, which involve immune and inflammatory responses. Immunological dysfunction significantly contributes to PD pathogenesis [28]. The SII quantifies the interaction between systemic inflammation and the immune response. The benefit of the SII lies in its ability to integrate three leukocyte subtypes, which can be easily calculated via peripheral blood platelet, neutrophil, and lymphocyte counts. Consequently, the SII serves as a straightforward and cost-effective indicator of inflammation. In daily clinical practice, many studies have confirmed that the SII can comprehensively reflect the body’s inflammatory status and has superior predictive value compared to other traditional inflammatory indicators [29,30]. As previously described, the SII, originally utilized in hepatocellular carcinoma, has been reported to significantly correlate with prognostic clinical outcomes [29]. Numerous clinical observation studies indicate that the SII is a reliable predictor of survival outcomes in various solid cancers, including cervical cancer [30], esophageal squamous cell carcinoma [31], and colorectal cancer [32]. Recent research has indicated that the SII is significantly linked to various non-neoplastic diseases, including stroke [33], metabolic syndrome [34], anemia [20], insulin resistance [35], prediabetes [35], depression [36], cognitive impairment [37], chronic kidney disease [38], rheumatoid arthritis [39], and isolated coronary artery ectasia [40]. Consequently, the SII has emerged as a simple, reliable, and minimally invasive biomarker with promising clinical applications.

Currently, the exact underlying mechanisms of the relationship between inflammation and PD are unclear. Research indicates that the inflammatory response may cause dopaminergic cell damage and death, contributing to the onset or progression of PD [13]. Chronic overexpression of IL-1β in the substantia nigra can lead to progressive dopaminergic cell death and glial activation [41]. Elevated TNF-α induces oxidative stress and cell death, particularly affecting dopaminergic neurons in PD [42]. Additionally, peripheral inflammation can activate microglia in the substantia nigra through the blood-brain barrier or the autonomic nervous system, such as the vagus nerve, triggering significant neurodegenerative processes in PD [43]. Furthermore, chronic inflammation in the olfactory or enteric system can promote pathological α-Syn aggregation [13]. The inflammatory response facilitates cell-to-cell transmission of the αSyn protein via IL-1β/IL-1 receptor 1-dependent signaling, resulting in cognitive impairments and motor dysfunction [13,44].

Although neutrophils, platelets, and lymphocytes are fundamental inflammation markers, they play a crucial role in the progression of PD. Chronic inflammation compromises the integrity of the BBB, allowing lymphocytes to infiltrate the CNS, exacerbating BBB disruption and attracting additional lymphocytes [13]. The infiltration of CD4+ and CD8+ T cells into the CNS leads to dopaminergic neurodegeneration in PD [45]. Research indicates that CD4+ T cells produce pro-inflammatory cytokines, including IFN-γ, TNF-α, and IL-17, which activate microglia to release neurotoxic factors such as reactive oxygen species (ROS) and glutamate, resulting in neuronal cell death [46,47]. CD8+ T cells initiate an autoimmune response against mitochondrial antigens, resulting in dopaminergic neurodegeneration [48]. On the other hand, Th17-mediated responses attract peripheral blood neutrophils to the target tissue, enhancing inflammation via degranulation [45]. Additionally, platelets play a crucial role in the inflammatory process. Platelets possess mitochondrial monoamine oxidase-B, an enzyme that mediates MPTP toxicity by forming MPPs, contributing to the development of PD [49].

The advantages of the present study are reflected in several aspects. Firstly, we utilized a nationally representative and adequately large sample of the U.S. population. Secondly, to improve result reliability, we applied appropriate weights and adjusted for known and potential confounding factors affecting the SII and PD relationship throughout the study. Finally, the restrictive cubic spline and smooth curve fitting method was used to investigate the potential nonlinear association between the SII and PD risk. Nonetheless, this study has certain limitations that need to be considered. First, the cross-sectional design inherently limits the ability to establish definitive causal relationships. Thus, further longitudinal research is necessary. Second, despite adjusting for multiple potential confounders, the association between the SII and PD risk may still be affected by residual confounders. Third, the NHANES identifies PD patients based on their use of anti-Parkinson drugs, which is similar to prior studies. This approach may have missed some cases of PD. In addition, the dataset used for this study does not include information on PD subtypes; therefore, we cannot further explore the relationship between systemic inflammation and specific PD subtypes. Furthermore, psychiatric diseases, antipsychotic drug use, and coffee consumption were not fully analyzed due to substantial missing data, which may weaken the findings. Future studies with more complete datasets are needed to explore their roles in systemic inflammation and PD risk. Finally, inflammatory diseases or infections may increase inflammation levels and introduce confounding factors into our findings. However, the data on these conditions were unavailable, which should be regarded as a shortcoming of the study.

## 5. Conclusions

Our results reveal that elevated SII values were associated with a higher incidence of PD, particularly among females. The SII may serve as a simple and straightforward approach to identify individuals with PD. Further prospective and experimental research is necessary to validate these results and clarify the underlying molecular mechanisms.

## Figures and Tables

**Table 1:** Weighted characteristics of the study population based on PD status.

Variable	Total	Non-Parkinson	Parkinson	*p*-Value
No.	30,638	30,222	416	
Age (year), mean + SD	58.34 ± 0.14	58.27 ± 0.14	63.02 ± 0.87	<0.0001
Gender, n (%)				0.01
Male	15,014 (47.28)	14,814 (47.40)	200 (38.18)	
Female	15,624 (52.72)	15,408 (52.60)	216 (61.82)	
Marital status, n (%)				<0.001
Married	17,850 (63.96)	17,640 (64.13)	210 (51.96)	
Unmarried	12,788 (36.04)	12,582 (35.87)	206 (48.04)	
Education level, n (%)				0.65
Less than high school	4047 (6.45)	4000 (6.45)	47 (6.17)	
High school	11,275 (34.45)	11,117 (34.42)	158 (36.86)	
More than high school	15,316 (59.10)	15,105 (59.13)	211 (56.96)	
Race, n (%)				<0.0001
White	13,710 (72.62)	13,439 (72.47)	271 (83.49)	
Black	6568 (9.82)	6513 (9.87)	55 (6.67)	
Other	10,360 (17.56)	10,270 (17.66)	90 (9.85)	
Smoke, n (%)				0.9
No	15,853 (51.79)	15,658 (51.79)	195 (51.41)	
Yes	14,785 (48.21)	14,564 (48.21)	221 (48.59)	
Stroke, n (%)				<0.0001
No	28,756 (95.34)	28,409 (95.48)	347 (84.88)	
Yes	1882 (4.66)	1813 (4.52)	69 (15.12)	
Hyperlipidemia, n (%)				0.41
No	6279 (20.02)	6208 (20.06)	71 (17.43)	
Yes	24,359 (79.98)	24,014 (79.94)	345 (82.57)	
Diabetes, n (%)				<0.001
No	24,450 (84.69)	24,158 (84.80)	292 (76.59)	
Yes	6188 (15.31)	6064 (15.20)	124 (23.41)	
Coronary heart disease				<0.001
No	28,661 (94.27)	28,299 (94.35)	362 (88.61)	
Yes	1977 (5.73)	1923 (5.65)	54 (11.39)	
Hypertension, n (%)				0.004
No	1368 (4.31)	1357 (4.34)	11 (1.66)	
Yes	29,270 (95.69)	28,865 (95.66)	405 (98.34)	
SII	5.63 ± 0.04	5.61 ± 0.04	7.44 ± 0.65	0.005
Red blood cell	4.66 ± 0.01	4.66 ± 0.01	4.53 ± 0.04	<0.001
White blood cell	7.19 ± 0.03	7.18 ± 0.03	7.58 ± 0.19	0.04
Alanine aminotransferase IU/L	24.66 ± 0.13	24.68 ± 0.13	22.92 ± 1.27	0.17
Aspartate aminotransferase IU/L	25.20 ± 0.12	25.20 ± 0.12	25.28 ± 1.19	0.95
Globulin g/dL	2.86 ± 0.01	2.86 ± 0.01	2.85 ± 0.03	0.74
Creatinine mg/dL	0.92 ± 0.00	0.92 ± 0.00	0.95 ± 0.02	0.07
Uric acid μmol/L	325.92 ± 0.76	325.97 ± 0.77	322.47 ± 5.40	0.52
Sodium mmol/L	139.51 ± 0.07	139.51 ± 0.07	139.50 ± 0.19	0.95
Calcium total mg/dL	9.40 ± 0.01	9.40 ± 0.01	9.35 ± 0.03	0.07

**Table 2:** Univariate logistic regression models of PD.

**Variables**	**OR (95% CI)**	***p* Value**
Age (years)	1.03 (1.02, 1.05)	<0.0001
Gender		
Male	Ref.	Ref.
Female	1.46 (1.09, 1.95)	0.01
Marital		
Married	Ref.	Ref.
Unmarried	1.65 (1.25, 2.19)	<0.001
Education level		
Less than high school	Ref.	Ref.
High school	1.12 (0.74, 1.69)	0.59
More than high school	1.01 (0.71, 1.42)	0.97
Race		
White	Ref.	Ref.
Black	0.59 (0.43, 0.80)	<0.001
Other	0.48 (0.36, 0.66)	<0.0001
Stroke		
No	Ref.	Ref.
Yes	3.76 (2.61, 5.42)	<0.0001
Hyperlipidemia		
No	Ref.	Ref.
Yes	1.19 (0.78, 1.80)	0.41
Diabetes		
No	Ref.	Ref.
Yes	1.70 (1.28, 2.27)	<0.001
Coronary heart disease		
No	Ref.	Ref.
Yes	2.14 (1.44, 3.18)	<0.001
Hypertension		
No	Ref.	Ref.
Yes	2.70 (1.33, 5.46)	0.01
Smoke		
No	Ref.	Ref.
Yes	1.02 (0.80, 1.29)	0.90
SII	1.07 (1.04, 1.10)	<0.0001
Red blood cell	0.58 (0.43, 0.78)	<0.001
White blood cell	1.01 (1.00, 1.02)	0.01
Alanine aminotransferase, IU/L	0.99 (0.97, 1.01)	0.31
Aspartate aminotransferase, IU/L	1.00 (0.99, 1.01)	0.94
Globulin g/dL	0.95 (0.71, 1.29)	0.75
Creatinine mg/dL	1.14 (1.04, 1.24)	0.004
Uric acid umol/L	1.00 (1.00, 1.00)	0.53
Sodium mmol/L	1.00 (0.95, 1.05)	0.95
Calcium total mg/dL	0.69 (0.46, 1.04)	0.08

**Table 3:** Multivariate logistic regression of relationship with SII and PD.

	Crude Model *		Model 1 *		Model 2 *		Model 3 *	
Character	95% CI	*p*	95% CI	*p*	95% CI	*p*	95% CI	*p*
Continuous	1.07 (1.04, 1.10)	<0.0001	1.06 (1.03, 1.09)	<0.001	1.06 (1.02, 1.09)	<0.001	1.05 (1.02, 1.09)	<0.001
SII quartiles								
Q1	Ref.		Ref.		Ref.		Ref.	
Q2	1.32 (0.90, 1.96)	0.1578	1.27 (0.86, 1.89)	0.2258	1.27 (0.86, 1.87)	0.2358	1.29 (0.87, 1.91)	0.2008
Q3	1.60 (1.18, 2.17)	0.0029	1.43 (1.04, 1.95)	0.0269	1.39 (1.02, 1.88)	0.0360	1.39 (1.02, 1.91)	0.0390
*p* for trend		0.0022		0.0239		0.0334		0.0377

* Crude model: does not contain any covariates. Model 1 * contains only age, education, sex, race, and marital status. Model 2 * contains age, education, sex, race, marital status, diabetes, hyperlipidemia, stroke, hypertension, coronary heart disease, and smoking status. Model 3 * includes all covariates (age, education, sex, race, marital status, diabetes, hyperlipidemia, stroke, hypertension, coronary heart disease, smoking status, red and white blood cell counts, alanine and aspartate aminotransferase levels, creatinine, globulin, uric acid, sodium, and calcium).

## Data Availability

The datasets utilized in this study are accessible through online repositories. The repository names and corresponding accession numbers can be accessed at: https://www.cdc.gov/nchs/nhanes/ (accessed on 1 December 2024). For additional questions, please contact the corresponding author directly.
